# Translation and Cross-Cultural Adaptation of The Cancer Dyspnoea Scale - CDS into Brazilian Portuguese

**DOI:** 10.1590/2317-1782/e20240259en

**Published:** 2025-10-24

**Authors:** Jaqueline Drigo da Fonseca, Renata Mendonça de Barros, Keiko Tanaka, Felipe Moreti, Mara Behlau

**Affiliations:** 1 Centro de Estudos da Voz - CEV - São Paulo (SP), Brasil.; 2 Hospital Anchieta, Complexo de Saúde de São Bernardo do Campo - CSSBC - São Bernardo do Campo (SP), Brasil.; 3 Department of Palliative Care, Tokyo Metropolitan Cancer and Infectious Diseases Center Komagome Hospital - Tokyo, Japan.; 4 Departamento de Fonoaudiologia, Faculdade de Filosofia e Ciências, Universidade Estadual Paulista “Júlio de Mesquita Filho” - Unesp - Marília (SP), Brasil.

**Keywords:** Dyspnea, Neoplasms, Self-Testing, Surveys and Questionnaires, Translating, Speech-Language Pathology

## Abstract

**Purpose:**

To translate and cross-culturally adapt The Cancer Dyspnoea Scale - CDS into Brazilian Portuguese.

**Methods:**

The original instrument in English was translated by a Brazilian Speech-Language Pathologist (SLP) and a Brazilian translator, both bilingual. The compiled version of the two translations was back-translated by a second SLP and a second translator, both bilingual, native English speakers, and fluent in Brazilian Portuguese. The final version was analyzed by a committee of experts, resulting in the final version of the *Escala de Dispneia Oncológica - CDS-Br*. It was pre-tested by applying the translated scale (with the additional option of responding “not applicable”) to 40 cancer patients with dyspnea complaints – 35 initial participants and another five to confirm subsequent adjustments.

**Results:**

None of the 35 initial study participants marked “not applicable” in the 12 scale items, but some questions were raised regarding the response keys, which were adapted after the committee of experts rediscussed them. Five new participants then responded to the scale to confirm the adjustments, without the need for further adaptations in the formulation of the items.

**Conclusion:**

The translated and cross-culturally adapted version for Brazilian Portuguese was called *Escala de Dispneia Oncológica - CDS-Br*. Adjustments were necessary during translation and cross-cultural adaptation to reflect the original version and be compatible with the target language and culture. The *Escala de Dispneia Oncológica - CDS-Br* proved to be an easy-to-apply and understand self-assessment tool for dyspnea in cancer patients.

## INTRODUCTION

Dyspnea can be defined as a subjective experience of respiratory discomfort^(1)^. This complex multidimensional symptom is difficult to interpret, as it varies in intensity according to the underlying disease and emotional state and may or may not be associated with other clinical changes^(2)^.

Several studies indicate that dyspnea is a very common symptom in patients with chronic and advanced diseases. Its prevalence can reach 50% of cancer patients, 74% of those with lung cancer, and 80% of those in the final stages of life. It can also be refractory to treatment, even if there is no lung involvement^(2-5)^.

Some studies have shown correlations between dyspnea and psychological state^(6-8)^, while others demonstrate an association with different etiologies and physical causes linked to the clinical condition^(4,9,10)^. In all these scenarios, diagnosis, oncological treatment, and disease progression may influence dyspnea in cancer patients^(8)^.

All these multidimensional factors suggest that dyspnea includes several sensations and mechanisms and is associated with decreased quality of life, progressing as the disease worsens^(11)^. This subjective, individual symptom requires appropriate self-assessment tools to understand possible etiologies and establish therapeutic strategies for dyspnea in the oncology population^(3,10)^.

The respiratory discomfort self-assessment instruments available for Brazilian Portuguese generally measure the presence of dyspnea, but do not focus on the specific changes in the oncological population. Moreover, they often assess patients under situations of exertion, which may be unfeasible for patients undergoing cancer treatment^(4,6)^.

The Cancer Dyspnoea Scale - CDS was developed^(6)^ as a brief self-assessment of dyspnea in cancer patients. Originally validated in English, it aims to understand multidimensional aspects based on self-assessment, which is particularly interesting due to the subjective nature of dyspnea. The CDS is an easy, short, and simple protocol that can be completed by patients, even if they are uncomfortable due to dyspnea. It can be applied even when the patient is bedridden, as it assesses dyspnea perceived in a normal situation, rather than under exertion^(6)^. The original version’s reliability and validity were confirmed in cancer patients, and it proved to be sensitive to clinical changes due to treatment or disease progression over time^(6)^.

There are no validated tools in Brazil for assessing dyspnea in cancer patients. Therefore, the translation, cross-cultural adaptation, and validation of this instrument are essential to aid in the diagnosis and define intervention strategies in this population, based on symptom self-assessment. This study aimed to translate and cross-culturally adapt the CDS self-assessment instrument^(6)^ into Brazilian Portuguese. These are essential initial phases for validating it in the Brazilian language and culture.

## METHODS

The cross-sectional study was approved by the Research Ethics Committee of the FMABC University Center, under approval number 6,584,015, of December 15, 2023, in accordance with Brazilian CNS Resolution 466/12. All participants provided their consent by signing an informed consent form.

The cross-cultural adaptation steps followed the literature recommendations for the stages of translation, synthesis, back-translation, review by the expert committee, and pre-testing^(12)^. A Speech-Language Pathologist (SLP) specialized in voice (T1) and a translator with no knowledge of the subject (T2), both native speakers of Brazilian Portuguese (target language) and fluent in English (source language), were invited to work independently on the initial translation. The researchers analyzed their translations and created a synthesized translated version (VCP). When the researchers disagreed, they opted for the choice that best fit Brazilian Portuguese, maintaining the concept of the original version. The consensus version was back-translated by a second SLP (R1) and a second translator with no knowledge of the subject (R2), both native speakers of English and fluent in Portuguese. A committee of five experts, including a bilingual Portuguese-English translator with extensive experience in scientific translations, a methodologist with a postgraduate degree in statistics and extensive experience in validating SLP self-assessment instruments, and three SLPs encompassing voice specialists, masters, and PhDs in Sciences, with expertise in oncological clinical practice and research – one of them is a senior with 47 years of experience, and two are progressing in their careers, with 27 and 18 years of experience. They compared the back-translation with the original to ensure semantic, conceptual, idiomatic, experiential, cultural, and operational equivalence. After these steps, a cross-culturally adapted version was pre-tested with 40 participants, following the guidelines of the COnsensus-based Standards for the selection of health Measurement INstruments (COSMIN)^(13)^.

The pre-test included subjects with confirmed diagnosis of head and neck cancer, over 18 years old, in clinical conditions to complete the questionnaires individually, and with self-reported dyspnea. The selection of patients with these characteristics was adapted to the current context after discussing it with the author of the original English version. Those with neurological or cognitive changes that could affect the instrument self-administration were excluded. Participants were recruited at *Hospital Anchieta*, a unit of the *Complexo de Saúde de São Bernardo do Campo - CSSBC*.

The *Escala de Dispneia Oncológica - CDS-Br* was applied with five response options, according to the original protocol, adding the option "not applicable" for situations that are culturally inapplicable or difficult for the participant to understand.

The data were analyzed descriptively and inferentially using SPSS 29.0 software. The significance level was set at 5% for inferential analyses.

The descriptive analysis calculated quantitative variables and measures of central tendency (mean and median), variability (standard deviation), and position (minimum, maximum, first, and third quartiles). The descriptive analysis of qualitative variables calculated absolute frequency and relative frequency percentage.

The proportion of two categories of a nominal qualitative variable was compared using the one-sample binomial test, with a reference proportion of 0.5. The observed and expected proportion for a nominal qualitative variable with multiple categories was compared using the one-sample chi-square test. The expected reference proportion was adopted as proportional to the number of categories in the response key.

## RESULTS

Altogether, 40 oncological patients with complaints of dyspnea participated in the study, with a mean age of 60.75 (standard deviation of 8.24, minimum of 39, and maximum of 76 years), 27 males (67.5%) and 13 females (32.5%), as shown in Table 1. There was a higher frequency of participants with complaints of dyspnea with an oncological diagnosis of laryngeal SCC (n=4; 10%), oropharyngeal SCC (n=4; 10%), who did not use a tracheostomy (n=24; 60%), who underwent surgery (n=24; 60%), who underwent radiotherapy (n=34; 85%), and who underwent chemotherapy (n=27; 67.5%).

**Table 1:** Descriptive analysis of the variables age, sex, oncological diagnosis, tracheostomy, surgery, radiotherapy and chemotherapy in oncological individuals with complaints of dyspnea

Variable and categories	n	%
Sex		
Male	27	67.50
Female	13	32.50
Oncological diagnosis		
Cystic adenoma of the parotid gland	1	2.50
Classic papillary thyroid carcinoma	1	2.50
Grade III squamous cell carcinoma of the right tonsillar cavity + right vocal fold paralysis	1	2.50
Undifferentiated carcinoma of the nasopharynx	1	2.50
Squamous cell carcinoma of the anterior floor of the mouth	3	7.50
Squamous cell carcinoma of the posterior floor of the mouth	1	2.50
Squamous cell carcinoma of the floor of the mouth and lateral border of the tongue	1	2.50
Squamous cell carcinoma of the base of the tongue	2	5.00
Squamous cell carcinoma of the border of the tongue	1	2.50
Squamous cell carcinoma of the glottis	3	7.50
Squamous cell carcinoma of the hypopharynx	1	2.50
Squamous cell carcinoma of the larynx	4	10.00
Squamous cell carcinoma of the tongue	3	7.50
Squamous cell carcinoma of the left tonsillar cavity	1	2.50
Squamous cell carcinoma of the oropharynx	4	10.00
Squamous cell carcinoma of the vocal fold	2	5.00
Squamous cell carcinoma of the pyriform sinus	1	2.50
Squamous cell carcinoma of the supraglottis	1	2.50
Squamous cell carcinoma of the vallecula with extension to the larynx and base of the tongue	1	2.50
Non-Hodgkin's lymphoma of large B cells of the oropharynx	1	2.50
Tongue dorsum neoplasm on the left	1	2.50
Hypopharyngeal neoplasm	1	2.50
Hypopharyngeal and laryngeal neoplasm	1	2.50
Soft palate neoplasm / 2^nd^ Squamous cell carcinoma of the floor of the mouth on the right / 3^rd^ Squamous cell carcinoma of the right retromolar region	1	2.50
Pyriform sinus neoplasm	1	2.50
Vagus paraganglioma	1	2.50
Tracheostomy		
No	24	60.00
Yes	16	40.00
Surgery		
No	16	40.00
Yes	24	60.00
Radiotherapy		
No	6	15.00
Yes	34	85.00
Chemotherapy		
No	13	32.50
Yes	27	67.50

Descriptive analysis

**Caption:** n = absolute frequency; % = relative frequency

The translators and experts discussed and adapted the CDS items. They initially diverged about the interpretation of the scale's wording, response keys, and specific items (5, 6, 7, 8, 9, 10, 11, and 12). The translators compared different linguistic and cultural approaches, reflecting on how each word and phrase could affect the participants' understanding and response. The expert committee adapted the verb tenses in items 10 and 12 in the final version of the scale after the second translation stage.

The process of translation, back-translation, and cross-cultural adaptation of the *Escala de Dispneia Oncológica - CDS-Br* is shown in Chart 1.

**Chart 1:** Process of translation and cross-cultural adaptation of The Cancer Dyspnoea Scale - CDS^(6)^ into Brazilian Portuguese, called *Escala de Dispneia Oncológica - CDS-Br*

**Items from the original English version**	**Translation into Brazilian Portuguese**	**Back-translation into English**	**Committee of SLP / Final translated and culturally adapted version**
The Cancer Dyspnoea Scale - CDS	*T1. Escala de Dispneia Oncológica*	R1. Oncological Dyspnea Scale	*Escala de Dispneia Oncológica - CDS-Br*
	*T2. Escala de Dispneia Oncológica*	R2. Cancer Dyspnea Scale	
	*VCP. Escala de Dispneia Oncológica*		
We would like to ask you about your breathlessness or difficulty in breathing. Please answer each question by circling only the numbers that best describes the breathing difficulty that you felt during the past few days. Base your response on your first impression.	*T1. Queremos compreender sua falta de ar ou dificuldade de respirar. Por favor, responda a cada pergunta assinalando o número que melhor descreve sua dificuldade de respirar, nos últimos dias. Responda de acordo com sua primeira impressão.*	R1. We want to ask about your shortness of breath or difficulty breathing. Please answer each question by marking the number that best describes your difficulty breathing in the past few days. Respond according to your first impression.	*Gostaríamos de lhe perguntar sobre sua falta de ar ou dificuldade de respirar. Por favor, responda a cada pergunta marcando o número que melhor descreve sua dificuldade de respirar, nos últimos dias. Responda de acordo com sua primeira impressão.*
	*T2. Gostaríamos de lhe perguntar sobre sua falta de ar ou dificuldade de respirar. Responda a cada pergunta fazendo um círculo em torno apenas dos números que melhor descrevem a dificuldade respiratória que você sentiu nos últimos dias. Baseie sua resposta em sua primeira impressão.*	R2. We want to ask you about your shortness of breath or difficulty breathing. Please answer each question by checking the number that better describes your difficulty breathing in the last few days. Answer according to your first impression.	
	*VCP. Queremos perguntar sobre sua falta de ar ou dificuldade de respirar. Por favor, responda a cada pergunta marcando o número que melhor descreve sua dificuldade de respirar, nos últimos dias. Responda de acordo com sua primeira impressão.*		
1 = Not at all	*T1.*	R1.	*1 = Não*
2 = A little	*1 = Nunca*	1 = Never	*2 = Um pouco*
3 = Somewhat	*2 = Raramente*	2 = Rarely	*3 = Mais ou menos*
4 = Considerably	*3 = Às vezes*	3 = Sometimes	*4 = Bastante*
5 = Very much	*4 = Muitas vezes*	4 = Often	*5 = Muito*
	*5 = Sempre*	5 = Always	
	*T2.*	R2.	
	*1 = De forma alguma*	1 = Never	
	*2 = Um pouco*	2 = Rarely	
	*3 = Um tanto*	3 = Sometimes	
	*4 = Consideravelmente*	4 = Often	
	*5 = Muito*	5 = Always	
	*VCP.*		
	*1 = Nunca*		
	*2 = Raramente*		
	*3 = Às vezes*		
	*4 = Muitas vezes*		
	*5 = Sempre*		
1. Can you inhale easily?	*T1. Você consegue inspirar com facilidade?*	R1. Can you inhale easily?	*1. Você consegue inspirar com facilidade?*
	*T2. Você consegue inspirar com facilidade?*	R2. Can you inhale easily?	
	*VCP. Você consegue inspirar com facilidade?*		
2. Can you exhale easily?	*T1. Você consegue expirar com facilidade?*	R1. Can you exhale easily?	*2. Você consegue expirar com facilidade?*
	*T2. Você consegue expirar com facilidade?*	R2. Can you exhale easily?	
	*VCP. Você consegue expirar com facilidade?*		
3. Can you breathe slowly?	*T1. Você consegue respirar lentamente?*	R1. Can you breathe slowly?	*3. Você consegue respirar lentamente?*
	*T2. Você consegue respirar lentamente?*	R2. Can you breathe slowly?	
	*VCP. Você consegue respirar lentamente?*		
4. Do you feel short of breath?	*T1. Você sente falta de ar?*	R1. Do you feel short of breath?	*4. Você sente falta de ar?*
	*T2. Você sente falta de ar?*	R2. Do you feel short of breath?	
	*VCP. Você sente falta de ar?*		
5. Do you feel breathing difficulty accompanied by palpitations and sweating?	*T1. Você sua e tem palpitações quando está difícil respirar?*	R1. Do you sweat and have palpitations when it is difficult to breathe?	*5. Você transpira e tem palpitações quando está difícil respirar?*
	*T2. Você sente dificuldade para respirar acompanhada de palpitações e sudorese?*	R2. Do you sweat and have palpitations when it is difficult to breathe?	
	*VCP. Você transpira e tem palpitações quando está difícil respirar?*		
6. Do you feel as if you are panting?	*T1. Você fica ofegante?*	R1. Do you feel panting?	*6. Você fica ofegante?*
	*T2. Você se sente como se estivesse ofegante?*	R2. Do you feel panting?	
	*VCP. Você fica ofegante?*		
7. Do you feel such breathing difficulty that you don't know what to do about it?	*T1. Você tem dificuldades respiratórias e fica sem saber o que fazer?*	R1. Do you have difficulty breathing and don't know what to do?	*7. Você tem dificuldade de respirar e fica sem saber o que fazer?*
	*T2. Você sente tanta dificuldade para respirar que não sabe o que fazer a respeito?*	R2. Do you have difficulty breathing and don't know what to do?	
	*VCP. Você tem dificuldade de respirar e fica sem saber o que fazer?*		
8.Do you feel your breath is shallow?	*T1. Você acha que sua respiração é superficial?*	R1. Do you feel that your breath is shallow?	*8. Você acha que sua respiração é superficial?*
	*T2. Você sente que sua respiração é rasa?*	R2. Do you think your breath is shallow?	
	*VCP. Você acha que sua respiração é superficial?*		
9.Do you feel your breathing may stop?	*T1. Você acha que pode parar de respirar?*	R1. Do you feel that your breathing may stop?	*9. Você sente que sua respiração pode se interromper?*
	*T2. Você sente que sua respiração pode parar?*	R2. Do you feel like your breathing might stop?	
	*VCP. Você sente que sua respiração pode se interromper?*		
10. Do you feel your airway has become narrower?	*T1. Você sente suas vias respiratórias estão mais fechadas?*	R1. Do you feel that your airways are narrowing?	*10. Você sente que suas vias respiratórias estão mais fechadas?*
	*T2. Você sente que suas vias aéreas ficaram mais estreitas?*	R2. Do you feel like your airways are narrowed?	
	*VCP. Você sente que suas vias respiratórias estão mais fechadas?*		
11. Do you feel as if you are drowning?	*T1. Você acha que pode se sufocar?*	R1. Do you feel that you may suffocate?	*11. Você acha que pode se sufocar?*
	*T2. Você sente como se estivesse se afogando?*	R2. Do you think you might suffocate?	
	*VCP. Você acha que pode se sufocar?*		
12. Do you feel as if something is stucking your airway?	*T1. Você sente que tem algo que prende a sua respiração?*	R1. Do you feel that something is blocking your breathing?	*12.Você sente que tem algo limitando sua respiração*
	*T2. Você sente como se algo estivesse preso em suas vias aéreas?*	R2. Do you feel like there is something holding your breath?	
	*VCP. Você sente que tem algo que prende a sua respiração?*		
Factor 1 - sense of effort	*T1.*	R1.	*Fator 1 - sensação de esforço*
Factor 2 - sense of anxiety	*Fator 1 - esforço*	Factor 1 - sensation of effort	
Factor 3 - sense of discomfort	*Fator 2 - ansiedade*	Factor 2 - sensation of anxiety	*Fator 2 - sensação de ansiedade*
Total dyspnoea	*Fator 3 - desconforto*	Factor 3 - sensation of discomfort	*Fator 3 - sensação de desconforto*
	*Dispneia total*	Total dyspnea	*Dispneia total*
	*T2.*	R2.	
	*Fator 1 - sensação de esforço*	Factor 1 - sensation of effort	
	*Fator 2 - sensação de ansiedade*	Factor 2 - sensation of anxiety	
	*Fator 3 - sensação de desconforto*	Factor 3 - sensation of discomfort	
	*Dispneia total*	Total dyspnea	
	*VCP.*		
	*Fator 1 - sensação de esforço*		
	*Fator 2 - sensação de ansiedade*		
	*Fator 3 - sensação de desconfortoDispneia total*		

**Caption:** T1 = English-Portuguese translator number 1; T2 = English-Portuguese translator number 2; VCP = Portuguese version of the consensus between the two translators 1 and 2; R1 = translator of back-translation 1 Portuguese into English; R2 = translator of back-translation 2 Portuguese into English

The need for consensus led to substantial adjustments, including changes in wording to ensure clarity and self-explanation, as well as in the formulation of response keys to ensure that the scale maintained its accuracy and clinical utility. Specific questions in scale items were revised for better cultural correspondence and global understanding, adapting terms and temporal contexts to facilitate a more accurate and comprehensive response from patients.

The adaptation and technical precision in the translation increase the sensitivity needed to consider cultural and linguistic differences, ensuring that the scale is efficient and understandable in different clinical and social contexts for Brazilian Portuguese.

Initially, the response keys for the translated scale were “not at all”, “a little”, “somewhat”, “considerably”, and “very much”. In the initial pretest with 35 patients, using the response categories of the original protocol, there were no questions about the content of the items, but rather about what was meant by “very much”, relating the word to a context of “excess”. Therefore, the same committee of experts with the five professionals rediscussed the issue, adjusting the response scale to “not at all”, “a little”, “somewhat”, “considerably”, and “a lot”. The final scale with the modified response key was applied to five other subjects, totaling 40 individuals in the study, and no difficulties were identified. No one marked “not applicable”, which reinforces this population’s easy recognition of dyspnea symptoms.

The original protocol presents three factors. Although no confirmatory analysis was performed, it is interesting to point out that the mean scores in the *Escala de Dispneia Oncológica - CDS-Br* were 8.15 for Factor 1 - Sense of effort, 4.3 for Factor 2 - Sense of anxiety, and 3.4 for Factor 3 - Sense of discomfort. The total mean dyspnea score was 15.85 (Table 2).

**Table 2:** Descriptive analysis of the factor scores of the *Escala de Dispneia Oncológica - CDS-Br* in oncologic individuals with complaints of dyspnea

Variable	Average	SD	Minimum	Maximum	1Q	Median	3Q
Factor 1 - Sense of effort	8.13	5.24	0.00	19.00	3.25	7.50	12.00
Factor 2 - Sense of anxiety	4.30	3.89	0.00	13.00	1.00	3.00	8.00
Factor 3 - Sense of discomfort	3.40	3.04	0.00	9.00	0.00	3.00	6.00
Total dyspnea	15.83	10.66	1.00	38.00	7.00	13.00	25.75

Descriptive analysis

**Caption:** SD = standard deviation; 1Q = first quartile; 3Q = third quartile

Table 3 shows that it was not possible to compare the proportion of responses from the usual response key of the *Escala de Dispneia Oncológica - CDS-Br* with the option “not applicable” for all items. All oncological patients with complaints of dyspnea who participated in the pretest marked one of the options of the usual response key of the *Escala de Dispneia Oncológica - CDS-Br*, not using “not applicable”.

**Table 3:** Descriptive analysis of the comparison of observed and obtained proportion for the comparison of usual response key and the non-applicable category for each item of the *Escala de Dispneia Oncológica - CDS-Br* in oncologic individuals with complaints of dyspnea

Variable and categories	n	%	Observed proportion	Test proportion
Item 1				
Answers 1-5	40	100.00	1.00	0.5
Item 2				
Answers 1-5	40	100.00	1.00	0.5
Item 3				
Answers 1-5	40	100.00	1.00	0.5
Item 4				
Answers 1-5	40	100.00	1.00	0.5
Item 5				
Answers 1-5	40	100.00	1.00	0.5
Item 6				
Answers 1-5	40	100.00	1.00	0.5
Item 7				
Answers 1-5	40	100.00	1.00	0.5
Item 8				
Answers 1-5	40	100.00	1.00	0.5
Item 9				
Answers 1-5	40	100.00	1.00	0.5
Item 10				
Answers 1-5	40	100.00	1.00	0.5
Item 11				
Answers 1-5	40	100.00	1.00	0.5
Item 12				
Answers 1-5	40	100.00	1.00	0.5

One-sample Binomial Test

**Caption:** n = absolute frequency; % = relative frequency

Table 4 compares the observed and expected proportion of each response option for each item of the *Escala de Dispneia Oncológica - CDS-Br*. There was no difference for item 10 (X^2^ = 2.500), whose question is, “Do you feel your airway has become narrower?”. This indicates a more random pattern of responses, with no tendency towards a single most frequent answer key for the question in the oncologic population.

**Table 4:** Descriptive analysis of the comparison of observed and obtained proportion for each category of the usual response key for each item of the *Escala de Dispneia Oncológica - CDS-Br* in oncologic individuals with complaints of dyspnea

	n Observed	n Expected	Standard	X^2^	df	p-value
Item 1				14.750	4	0.005
1	2	8.0	-6.0			
2	3	8.0	-5.0			
3	10	8.0	2.0			
4	10	8.0	2.0			
5	15	8.0	7.0			
Total	40					
Item 2				15.500	4	0.004
1	1	8.0	-7.0			
2	4	8.0	-4.0			
3	9	8.0	1.0			
4	11	8.0	3.0			
5	15	8.0	7.0			
Total	40					
Item 3				16.750	4	0.002
1	1	8.0	-7.0			
2	4	8.0	-4.0			
3	9	8.0	1.0			
4	10	8.0	2.0			
5	16	8.0	8.0			
Total	40					
Item 4				13.000	4	0.011
1	11	8.0	3.0			
2	13	8.0	5.0			
3	2	8.0	-6.0			
4	11	8.0	3.0			
5	3	8.0	-5.0			
Total	40					
Item 5				26.750	4	0.000
1	18	8.0	10.0			
2	13	8.0	5.0			
3	6	8.0	-2.0			
4	2	8.0	-6.0			
5	1	8.0	-7.0			
Total	40					
Item 6				17.000	4	0.002
1	6	8.0	-2.0			
2	18	8.0	10.0			
3	4	8.0	-4.0			
4	8	8.0	0.0			
5	4	8.0	-4.0			
Total	40					
Item 7				16.750	4	0.002
1	17	8.0	9.0			
2	5	8.0	-3.0			
3	6	8.0	-2.0			
4	10	8.0	2.0			
5	2	8.0	-6.0			
Total	40					
Item 8				27.000	4	0.000
1	21	8.0	13.0			
2	3	8.0	-5.0			
3	5	8.0	-3.0			
4	6	8.0	-2.0			
5	5	8.0	-3.0			
Total	40					
Item 9				41.250	4	0.000
1	24	8.0	16.0			
2	2	8.0	-6.0			
3	5	8.0	-3.0			
4	6	8.0	-2.0			
5	3	8.0	-5.0			
Total	40					
Item 10				2.500	4	0.645
1	11	8.0	3.0			
2	7	8.0	-1.0			
3	5	8.0	-3.0			
4	9	8.0	1.0			
5	8	8.0	0.0			
Total	40					
Item 11				28.000	4	0.000
1	21	8.0	13.0			
2	7	8.0	-1.0			
3	5	8.0	-3.0			
4	5	8.0	-3.0			
5	2	8.0	-6.0			
Total	40					
Item 12				9.500	4	0.050
1	15	8.0	7.0			
2	5	8.0	-3.0			
3	4	8.0	-4.0			
4	7	8.0	-1.0			
5	9	8.0	1.0			
Total	40					

One-sample Chi-Square Test

**Caption:** n = absolute frequency; X^2^ = chi-square; df = degrees of freedom

The final makeup of the translated and cross-culturally adapted Brazilian version of the CDS^(6)^, called *Escala de Dispneia Oncológica - CDS-Br* (Appendix 1), has 12 items, like the original protocol.

## DISCUSSION

Dyspnea is a subjective experience of respiratory discomfort consisting of qualitatively distinct sensations that vary in intensity. It is one of the most common and distressing symptoms in cancer patients, aggravated by other related symptoms, such as fatigue and anxiety, causing functional limitation^(8)^. Several functions can be significantly impaired due to respiratory changes, which are directly caused by dyspnea or by its impact on prognostic evolution^(6,8)^. Dyspnea can interfere with vocal production due to changes in respiratory flow, impairing the quality of communication. In all possible scenarios, dyspnea and its morbidities can negatively compromise the quality of life of cancer patients^(7)^.

Due to the complexity and subjectivity of dyspnea, health professionals must be attentive to the management of the condition to ensure an appropriate approach and define the correct indication for interventions on related aspects that directly interfere with the respiratory condition. Self-assessment protocols help professionals to better understand the impact of dyspnea on patients and help patients clearly understand how this change has affected their well-being^(6)^. There are no self-assessment dyspnea scales or questionnaires in Brazil specifically for cancer patients, considering the multidimensional factors of dyspnea.

The CDS^(6)^ is a brief self-assessment questionnaire with three factors and 12 items. It is the first scale to address the multidimensional nature of dyspnea in cancer patients, focusing directly on the experience of dyspnea itself, rather than assessing only the physical exertion that can trigger it. It is a unique, self-administered, validated, and proven reliable tool for assessing dyspnea in cancer patients. The total and subfactor CDS scores reflect the intensity of dyspnea, and each subfactor captures different aspects of dyspnea, correlating with physical and psychological status^(6)^.

The initial phase of the questionnaire validation process encompasses translation and cross-cultural adaptation. These are essential to enable the use of this instrument in a language and culture different from those in which it was originally designed and must follow guidelines that ensure the scientific rigor of this procedure^(12)^. The experts modified scale questions (along with the marking items) during the synthesis and consensus phases to make them clearer and simpler and to ensure that they were understandable for different social and cultural groups in Brazil. It was necessary to simplify some questions and adjust terms that did not affect the general meaning of the sentences when compared to the original instrument.

The pretest phase found that all scale questions were understandable. No participant left a question unanswered, questioned its meaning, or used “not applicable” when it was available. However, there was room for discussion regarding the last option on the originally proposed answer key (“not at all”, “a little”, “somewhat”, “considerably”, and “very much”). Some participants mentioned that “very much” could be a factor for change, so the experts rediscussed the scale, which was adjusted to the proposed terms. The new participants using this self-assessment version had no questions.

A statistically significant difference was noted in the proportion of each response option in all items, except for item 10 (“Do you feel your airway has become narrower?”). Due to the participants’ oncological diagnoses, the pattern of responses can be related to the similarity among subjects in the group. Regarding item 10, this question may be affected by the participant’s respiratory environment. The study included patients not using tracheostomy and using protective tracheostomy, tracheostomy due to airway obstruction, and permanent tracheostomy. The respiratory pathway may have contributed to the pattern of responses to this item, demonstrating the effectiveness of using the scale in clinical practice for different types of patients, since it suggests that the multiple dimensions of dyspnea overlap in a complex way and are closely related^(6)^.

The application of the CDS-Br proved to be feasible and practical in a clinical setting. It is simple and easy for cancer patients with dyspnea to answer, which are crucial characteristics of the instrument^(6)^.

The cross-cultural adaptation carried out in this study resulted in the version of the *Escala de Dispneia Oncológica - CDS-Br* translated and cross-culturally adapted to Brazilian Portuguese, offering a unique instrument for cancer patients’ self-assessment of dyspnea.

## CONCLUSION

The translated and cross-culturally adapted version for Brazilian Portuguese was called *Escala de Dispneia Oncológica - CDS-Br*. Adjustments were necessary during translation and cross-cultural adaptation to make it applicable to Brazilian Portuguese, reflecting the original version. The *Escala de Dispneia Oncológica - CDS-Br* proved to be an easy-to-use and understand self-assessment instrument for dyspnea in cancer patients.

## Appendix 1 Escala de Dispneia Oncológica - CDS-Br, translated and culturally adapted version or The Cancer Dyspnoea Scale - CDS ^(6)^

**Table:**

**ESCALA DE DISPNEIA ONCOLÓGICA - CDS-Br**					
Gostaríamos de lhe perguntar sobre sua falta de ar ou dificuldade de respirar. Por favor, responda a cada pergunta marcando o número que melhor descreve sua dificuldade de respirar, nos últimos dias. Responda de acordo com sua primeira impressão.					
	Não	Um pouco	Mais ou menos	Bastante	Muito
1. Você consegue inspirar com facilidade?	1	2	3	4	5
2. Você consegue expirar com facilidade?	1	2	3	4	5
3. Você consegue respirar lentamente?	1	2	3	4	5
4. Você sente falta de ar?	1	2	3	4	5
5. Você transpira e tem palpitações quando está difícil respirar?	1	2	3	4	5
6. Você fica ofegante?	1	2	3	4	5
7. Você tem dificuldade de respirar e fica sem saber o que fazer?	1	2	3	4	5
8. Você acha que sua respiração é superficial?	1	2	3	4	5
9. Você sente que sua respiração pode se interromper?	1	2	3	4	5
10. Você sente que suas vias respiratórias estão mais fechadas?	1	2	3	4	5
11. Você acha que pode se sufocar?	1	2	3	4	5
12. Você sente que tem algo limitando sua respiração	1	2	3	4	5
**Como calcular os resultados**					
**1.** Some as pontuações de cada fator.					
Fator 1 = (itens 4 + 6 + 8 + 10 + 12) – 5 = **sensação de esforço**					
Fator 2 = (itens 5 + 7 + 9 + 11) – 4 = **sensação de ansiedade**					
Fator 3 = 15 – (itens 1 + 2 + 3) = **sensação de desconforto**					
**2.** Some a pontuação total de cada fator = **dispneia total**					
As subtrações servem para fazer ajustes para 0 corresponder ao estado de ausência de dispneia.					
