# Supplementary material for: Translation and Cross-Cultural Adaptation of The Cancer Dyspnoea Scale - CDS into Brazilian Portuguese
Source: Codas. 2025 Oct 24;37(5):e20240259. doi: 10.1590/2317-1782/e20240259en (PMC12617282; doi:10.1590/2317-1782/e20240259en)
Supplement: Translation

# Translation: Tradução e adaptação transcultural da *The Cancer Dyspnoea Scale - CDS* para o Português Brasileiro

**DOI:** 10.1590/2317-1782/e20240259pt

**Authors:** Jaqueline Drigo da Fonseca, Renata Mendonça de Barros, Keiko Tanaka, Felipe Moreti, Mara Behlau

**Affiliations:** 1 Centro de Estudos da Voz - CEV - São Paulo (SP), Brasil.; 2 Hospital Anchieta, Complexo de Saúde de São Bernardo do Campo - CSSBC - São Bernardo do Campo (SP), Brasil.; 3 Department of Palliative Care, Tokyo Metropolitan Cancer and Infectious Diseases Center Komagome Hospital - Tokyo, Japan.; 4 Departamento de Fonoaudiologia, Faculdade de Filosofia e Ciências, Universidade Estadual Paulista “Júlio de Mesquita Filho” - Unesp - Marília (SP), Brasil.

**Keywords:** Dispneia, Neoplasias, Autoteste, Inquéritos e Questionários, Tradução, Fonoaudiologia

## Abstract

**Objetivo:**

Realizar a tradução e adaptação transcultural da *The Cancer Dyspnoea Scale - CDS* para o Português Brasileiro (PB).

**Método:**

O instrumento original em inglês foi traduzido por um fonoaudiólogo brasileiro e um tradutor brasileiro, ambos bilíngues. A versão compilada das duas traduções foi retrotraduzida por um segundo fonoaudiólogo e um segundo tradutor, ambos bilíngues, nativos do inglês e fluentes em Português Brasileiro. A versão final foi analisada por comitê de especialistas, elaborando-se uma versão final da Escala de Dispneia Oncológica - CDS-Br. Foi realizado pré-teste com a aplicação da escala traduzida, acrescida da opção de resposta “não aplicável” em 40 sujeitos oncológicos com queixa de dispneia, sendo 35 participantes iniciais e cinco para confirmação de ajustes posteriores.

**Resultados:**

Dos 35 participantes iniciais do estudo, nenhum assinalou a opção “não aplicável” para os 12 itens da escala, porém foram observados questionamentos sobre as chaves de resposta, que foram rediscutidos pelo comitê de especialistas e adaptados. Em seguida, cinco novos participantes responderam a escala para a confirmação dos ajustes, sem necessidade de novas adaptações na formulação dos itens.

**Conclusão:**

A versão traduzida e transculturalmente adaptada para o Português Brasileiro foi denominada Escala de Dispneia Oncológica - CDS-Br. No processo de tradução e adaptação transcultural foram necessários ajustes para que reflita a versão original e seja compatível com a língua e cultura alvo. A Escala de Dispneia Oncológica - CDS-Br mostrou ser um instrumento de fácil aplicação e compreensão para a autoavaliação da dispneia de pacientes oncológicos.

## INTRODUÇÃO

A dispneia pode ser definida como uma experiência subjetiva de desconforto respiratório^(1)^. É um sintoma multidimensional complexo e de difícil interpretação, visto que varia de intensidade de acordo com a doença subjacente e estado emocional e pode estar ou não associado a outras alterações clínicas^(2)^.

Diversos estudos apontam para a dispneia como um sintoma muito frequente em pacientes com doenças crônicas e avançadas. Sua prevalência pode chegar a 50% dos pacientes com câncer, atingindo 74% nos pacientes com câncer de pulmão e 80% nesses quando em fase final de vida, podendo ser um sintoma refratário a tratamento, mesmo que não haja nenhum comprometimento pulmonar^(2-5)^.

Alguns estudos têm apontado correlações entre dispneia e estado psicológico^(6-8)^ enquanto outros demonstram associação com diferentes etiologias e causas físicas associados ao quadro clínico^(4,9,10)^. Em todos esses cenários a dispneia pode sofrer influência do diagnóstico, tratamento oncológico ou avanço da doença em pacientes com câncer^(8)^.

Todos esses fatores multidimensionais sugerem que a dispneia inclui diversas sensações e mecanismos e está associada à diminuição da qualidade de vida, sendo progressiva na medida do agravo da doença^(11)^. Devido ao caráter subjetivo e individual desse sintoma, é necessário a implementação de ferramentas de autoavaliação apropriadas para entender possíveis etiologias e estabelecer estratégias terapêuticas para dispneia na população oncológica^(3,10)^.

Os instrumentos de autoavaliação de desconforto respiratórios disponíveis para o Português Brasileiro mensuram de modo geral a presença de dispneia, mas não focam nas alterações específicas da população oncológica e, por diversas vezes, avaliam os pacientes sob situações de esforço, exigência que pode ser inviável ao paciente em tratamento de câncer^(4,6)^.

A *The Cancer Dyspnoea Scale - CDS* foi desenvolvida^(6)^ como uma breve autoavaliação da dispneia em pacientes com câncer. Validada originalmente em inglês, tem como objetivo compreender aspectos multidimensionais a partir de uma autoavaliação, o que é particularmente interessante pelo caráter subjetivo da dispneia. O CDS é um protocolo fácil, curto e simples o suficiente para ser preenchido pelos pacientes, mesmo que desconfortáveis devido a própria dispneia. Pelo fato de avaliar a dispneia percebida em situação habitual, e não sob esforço, pode ser aplicado mesmo com o paciente acamado^(6)^. Na versão original, sua confiabilidade e validade foram confirmadas em pacientes com câncer e a ferramenta se mostrou sensível às alterações clínicas devido ao tratamento ou progressão da doença ao longo do tempo^(6)^.

Não existem ferramentas validadas no Brasil para a avaliação de dispneia em pacientes oncológicos e, desta forma, torna-se essencial a tradução, adaptação transcultural e validação desse instrumento para auxiliar no diagnóstico e definir estratégias de intervenção nessa população, a partir da autoavaliação do sintoma. O presente estudo tem o objetivo de traduzir e adaptar transculturalmente o instrumento de autoavaliação *The Cancer Dyspnoea Scale - CDS* ^(6)^ para o Português Brasileiro, fases iniciais essenciais para validação na língua e cultura brasileira.

## MÉTODO

O estudo de desenho transversal recebeu a aprovação do Comitê de Ética em Pesquisa do Centro Universitário FMABC, sob parecer 6.584.015 de 15 de dezembro de 2023, em conformidade com a Resolução CNS 466/12. Todos os participantes forneceram seu consentimento por meio da assinatura do Termo de Consentimento Livre e Esclarecido - TCLE.

As etapas de adaptação transcultural seguiram as recomendações da literatura para as etapas de tradução, síntese, retrotradução, revisão pelo comitê de especialistas e pré-teste^(12)^. Para a tradução inicial, um fonoaudiólogo especializado em voz (T1) e um tradutor sem conhecimento no tema (T2), ambos nativos do Português Brasileiro (idioma alvo) e fluentes em inglês (idioma fonte), foram convidados a trabalhar de forma independente. Suas traduções foram analisadas pelos pesquisadores e uma versão de síntese traduzida foi criada (VCP). Quando houve discordância, os pesquisadores optaram pela escolha que melhor se ajustava ao Português Brasileiro, mantendo o conceito da versão original. A versão consensual foi retrotraduzida por um segundo fonoaudiólogo (R1) e um segundo tradutor sem conhecimento sobre o tema (R2), ambos nativos do inglês e fluentes em Português. Um comitê de cinco especialistas, incluindo um tradutor bilíngue português-inglês com bastante experiência em traduções científicas, um metodologista com formação em nível de pós-graduação em estatística e bastante experiência em processos de validações de instrumentos de autoavaliação na Fonoaudiologia e três fonoaudiólogos com Especialistas em Voz, mestres e doutores em Ciências e expertise de atuação clínica e em pesquisa na área de Oncologia, sendo um sênior com 47 anos de atuação, e dois em desenvolvimento de carreira: um com 27 anos de atuação e outro com 18 anos, comparou a retrotradução com o original para garantir equivalência semântica, conceitual, idiomática, experiencial, cultural e operacional. Após essas etapas, uma versão adaptada transculturalmente foi submetida ao pré-teste com 40 participantes, conforme as diretrizes do *COnsensus-based Standards for the selection of health Measurement INstruments* (COSMIN)^(13)^.

O pré-teste incluiu sujeitos com diagnóstico confirmado de câncer de cabeça e pescoço, maiores de 18 anos, em condições clínicas para completar os questionários de forma individual e queixa autorreferida de dispneia. A escolha de pacientes com essas características foi adaptada ao contexto atual após discussão com o autor da versão original em inglês. Foram excluídos aqueles com alterações neurológicas ou cognitivas que pudessem afetar a autoaplicação dos instrumentos. Os participantes foram recrutados no Hospital Anchieta, unidade do Complexo de Saúde de São Bernardo do Campo - CSSBC.

A Escala de Dispneia Oncológica - CDS-Br foi aplicada com cinco opções de resposta, conforme o protocolo original, com a adição da opção "Não aplicável" para situações culturalmente não aplicáveis ou de difícil compreensão para o participante.

Os dados foram analisados de forma descritiva e inferencial utilizando-se o software SPSS 29.0. Foi considerado um nível de significância de 5% para as análises inferenciais.

Na análise descritiva das variáveis quantitativas foram calculadas as medidas de tendência central (média e mediana), variabilidade (desvio-padrão) e posição (mínimo, máximo, primeiro e terceiro quartis). Na análise descritiva das variáveis qualitativas foram calculadas a frequência absoluta e a frequência relativa percentual.

A comparação da proporção de duas categorias de uma variável qualitativa nominal foi realizada por meio do Teste Binominal de uma amostra, adotando-se a proporção de referência de 0,5. A comparação da proporção observada e esperada para uma variável qualitativa nominal de múltiplas categorias foi realizada por meio do Teste Qui-Quadrado de uma amostra. Adotou-se a proporção de referência esperada proporcional ao número de categorias da chave de resposta.

## RESULTADOS

Participaram do estudo total 40 indivíduos oncológicos com queixa de dispneia, com média de idade de 60,75 (desvio padrão de 8,24; idade mínima 39 anos e idade máxima 76 anos), sendo 27 do sexo masculino (67,5%) e 13 do sexo feminino (32,5%), conforme mostra a Tabela 1. Houve maior frequência de indivíduos oncológicos com queixa de dispneia com diagnóstico oncológico de CEC de laringe (n=4; 10%), e de CEC de orofaringe (n=4; 10%), que não faziam uso de traqueostomia (n=24; 60%), que realizaram cirurgia (n=24; 60%), que realizaram radioterapia (n=34; 85%), e que realizaram quimioterapia (n=27; 67,5%).

**Tabela 1:** Análise descritiva das variáveis idade, sexo, diagnóstico oncológico, traqueostomia, cirurgia, radioterapia e quimioterapia em indivíduos oncológicos com queixa de dispneia

Variável e categorias	n	%
Sexo		
Masculino	27	67,50
Feminino	13	32,50
Diagnóstico oncológico		
Adenoma cístico de parótida	1	2,50
Carcinoma papilífero clássico de tireoide	1	2,50
Carcinoma epidermoide grau III de loja tonsilar direita + paralisia de prega vocal direita	1	2,50
Carcinoma indiferenciado de nasofaringe	1	2,50
Carcinoma espinocelular de assoalho anterior	3	7,50
Carcinoma espinocelular de assoalho posterior	1	2,50
Carcinoma espinocelular de assoalho e borda lateral de língua	1	2,50
Carcinoma espinocelular de base de língua	2	5,00
Carcinoma espinocelular de borda de língua	1	2,50
Carcinoma espinocelular de glote	3	7,50
Carcinoma espinocelular de hipofaringe	1	2,50
Carcinoma espinocelular de laringe	4	10,00
Carcinoma espinocelular de língua	3	7,50
Carcinoma espinocelular de loja tonsilar esquerda	1	2,50
Carcinoma espinocelular de orofaringe	4	10,00
Carcinoma espinocelular de prega vocal	2	5,00
Carcinoma espinocelular de seio piriforme	1	2,50
Carcinoma espinocelular de supraglote	1	2,50
Carcinoma espinocelular de valécula com extensão para laringe e base de língua	1	2,50
Linfoma Não Hodgkin de grandes células B de orofaringe	1	2,50
Neoplasia de dorso de língua à esquerda	1	2,50
Neoplasia de hipofaringe	1	2,50
Neoplasia de hipofaringe e laringe	1	2,50
Neoplasia de palato mole / 2° Carcinoma espinocelular de assoalho de boca à direita / 3° Carcinoma espinocelular região retromolar direita	1	2,50
Neoplasia de seio piriforme	1	2,50
Paraganglioma de vago	1	2,50
Traqueostomia		
Não	24	60,00
Sim	16	40,00
Cirurgia		
Não	16	40,00
Sim	24	60,00
Radioterapia		
Não	6	15,00
Sim	34	85,00
Quimioterapia		
Não	13	32,50
Sim	27	67,50

Análise descritiva

**Legenda:** n = frequência absoluta; % = frequência relativa

Os itens da *The Cancer Dyspnoea Scale - CDS* passaram por um processo de discussão e adaptação entre os tradutores e especialistas. Inicialmente, houve discordâncias na interpretação do enunciado da escala, nas chaves de resposta e nos itens específicos (5, 6, 7, 8, 9, 10, 11 e 12). Os tradutores confrontaram diferentes abordagens linguísticas e culturais, refletindo sobre como cada palavra e frase poderiam afetar a compreensão e resposta dos participantes. A versão final da escala foi adaptada pelo comitê de especialistas, após a segunda etapa de tradução, nos itens 10 e 12, com relação ao tempo verbal.

O processo de tradução, retrotradução e adaptação transcultural da Escala de Dispneia Oncológica - CDS-Br pode ser encontrado no Quadro 1.

**Quadro 1:** Processo de tradução e adaptação transcultural da *The Cancer Dyspnoea Scale - CDS* ^(6)^ para o português brasileiro, chamada de Escala de Dispneia Oncológica - CDS-Br

Itens da versão original em inglês	Tradução para o português brasileiro	Retrotradução para o inglês	Comitê de fonoaudiólogos especialistas / Versão final traduzida e culturalmente adaptada
*The Cancer Dyspnoea Scale - CDS*	T1. Escala de Dispneia Oncológica	*R1. Oncological Dyspnea Scale*	Escala de Dispneia Oncológica - CDS-Br
	T2. Escala de Dispneia Oncológica	*R2. Cancer Dyspnea Scale*	
	VCP. Escala de Dispneia Oncológica		
*We would like to ask you about your breathlessness or difficulty in breathing. Please answer each question by circling only the numbers that best describes the breathing difficulty that you felt during the past few days. Base your response on your first impression.*	T1. Queremos compreender sua falta de ar ou dificuldade de respirar. Por favor, responda a cada pergunta assinalando o número que melhor descreve sua dificuldade de respirar, nos últimos dias. Responda de acordo com sua primeira impressão.	*R1. We want to ask about your shortness of breath or difficulty breathing. Please answer each question by marking the number that best describes your difficulty breathing in the past few days. Respond according to your first impression.*	Gostaríamos de lhe perguntar sobre sua falta de ar ou dificuldade de respirar. Por favor, responda a cada pergunta marcando o número que melhor descreve sua dificuldade de respirar, nos últimos dias. Responda de acordo com sua primeira impressão.
	T2. Gostaríamos de lhe perguntar sobre sua falta de ar ou dificuldade de respirar. Responda a cada pergunta fazendo um círculo em torno apenas dos números que melhor descrevem a dificuldade respiratória que você sentiu nos últimos dias. Baseie sua resposta em sua primeira impressão.	*R2. We want to ask you about your shortness of breath or difficulty breathing. Please answer each question by checking the number that better describes your difficulty breathing in the last few days. Answer according to your first impression.*	
	VCP. Queremos perguntar sobre sua falta de ar ou dificuldade de respirar. Por favor, responda a cada pergunta marcando o número que melhor descreve sua dificuldade de respirar, nos últimos dias. Responda de acordo com sua primeira impressão.		
*1 = Not at all*	T1.	*R1.*	1 = Não
*2 = A little*	1 = Nunca	*1 = Never*	2 = Um pouco
*3 = Somewhat*	2 = Raramente	*2 = Rarely*	3 = Mais ou menos
*4 = Considerably*	3 = Às vezes	*3 = Sometimes*	4 = Bastante
*5 = Very much*	4 = Muitas vezes	*4 = Often*	5 = Muito
	5 = Sempre	*5 = Always*	
	T2.	*R2.*	
	1 = De forma alguma	*1 = Never*	
	2 = Um pouco	*2 = Rarely*	
	3 = Um tanto	*3 = Sometimes*	
	4 = Consideravelmente	*4 = Often*	
	5 = Muito	*5 = Always*	
	VCP.		
	1 = Nunca		
	2 = Raramente		
	3 = Às vezes		
	4 = Muitas vezes		
	5 = Sempre		
*1. Can you inhale easily?*	T1. Você consegue inspirar com facilidade?	*R1. Can you inhale easily?*	1. Você consegue inspirar com facilidade?
	T2. Você consegue inspirar com facilidade?	*R2. Can you inhale easily?*	
	VCP. Você consegue inspirar com facilidade?		
*2. Can you exhale easily?*	T1. Você consegue expirar com facilidade?	*R1. Can you exhale easily?*	2. Você consegue expirar com facilidade?
	T2. Você consegue expirar com facilidade?	*R2. Can you exhale easily?*	
	VCP. Você consegue expirar com facilidade?		
*3. Can you breathe slowly?*	T1. Você consegue respirar lentamente?	*R1. Can you breathe slowly?*	3. Você consegue respirar lentamente?
	T2. Você consegue respirar lentamente?	*R2. Can you breathe slowly?*	
	VCP. Você consegue respirar lentamente?		
*4. Do you feel short of breath?*	T1. Você sente falta de ar?	*R1. Do you feel short of breath?*	4. Você sente falta de ar?
	T2. Você sente falta de ar?	*R2. Do you feel short of breath?*	
	VCP. Você sente falta de ar?		
*5. Do you feel breathing difficulty accompanied by palpitations and sweating?*	T1. Você sua e tem palpitações quando está difícil respirar?	*R1. Do you sweat and have palpitations when it is difficult to breathe?*	5. Você transpira e tem palpitações quando está difícil respirar?
	T2. Você sente dificuldade para respirar acompanhada de palpitações e sudorese?	*R2. Do you sweat and have palpitations when it is difficult to breathe?*	
	VCP. Você transpira e tem palpitações quando está difícil respirar?		
*6. Do you feel as if you are panting?*	T1. Você fica ofegante?	*R1. Do you feel panting?*	6. Você fica ofegante?
	T2. Você se sente como se estivesse ofegante?	*R2. Do you feel panting?*	
	VCP. Você fica ofegante?		
*7. Do you feel such breathing difficulty that you don't know what to do about it?*	T1. Você tem dificuldades respiratórias e fica sem saber o que fazer?	*R1. Do you have difficulty breathing and don't know what to do?*	7. Você tem dificuldade de respirar e fica sem saber o que fazer?
	T2. Você sente tanta dificuldade para respirar que não sabe o que fazer a respeito?	*R2. Do you have difficulty breathing and don't know what to do?*	
	VCP. Você tem dificuldade de respirar e fica sem saber o que fazer?		
*8.Do you feel your breath is shallow?*	T1. Você acha que sua respiração é superficial?	*R1. Do you feel that your breath is shallow?*	8. Você acha que sua respiração é superficial?
	T2. Você sente que sua respiração é rasa?	*R2. Do you think your breath is shallow?*	
	VCP. Você acha que sua respiração é superficial?		
*9.Do you feel your breathing may stop?*	T1. Você acha que pode parar de respirar?	*R1. Do you feel that your breathing may stop?*	9. Você sente que sua respiração pode se interromper?
	T2. Você sente que sua respiração pode parar?	*R2. Do you feel like your breathing might stop?*	
	VCP. Você sente que sua respiração pode se interromper?		
*10. Do you feel your airway has become narrower?*	T1. Você sente suas vias respiratórias estão mais fechadas?	*R1. Do you feel that your airways are narrowing?*	10. Você sente que suas vias respiratórias estão mais fechadas?
	T2. Você sente que suas vias aéreas ficaram mais estreitas?	*R2. Do you feel like your airways are narrowed?*	
	VCP. Você sente que suas vias respiratórias estão mais fechadas?		
*11. Do you feel as if you are drowning?*	T1. Você acha que pode se sufocar?	*R1. Do you feel that you may suffocate?*	11. Você acha que pode se sufocar?
	T2. Você sente como se estivesse se afogando?	*R2. Do you think you might suffocate?*	
	VCP. Você acha que pode se sufocar?		
*12. Do you feel as if something is stucking your airway?*	T1. Você sente que tem algo que prende a sua respiração?	*R1. Do you feel that something is blocking your breathing?*	12.Você sente que tem algo limitando sua respiração
	T2. Você sente como se algo estivesse preso em suas vias aéreas?	*R2. Do you feel like there is something holding your breath?*	
	VCP. Você sente que tem algo que prende a sua respiração?		
*Factor 1 - sense of effort*	T1.	*R1.*	Fator 1 - sensação de esforço
*Factor 2 - sense of anxiety*	Fator 1 - esforço	*Factor 1 - sensation of effort*	
*Factor 3 - sense of discomfort*	Fator 2 - ansiedade	*Factor 2 - sensation of anxiety*	Fator 2 - sensação de ansiedade
*Total dyspnoea*	Fator 3 - desconforto	*Factor 3 - sensation of discomfort*	Fator 3 - sensação de desconforto
	Dispneia total	*Total dyspnea*	Dispneia total
	T2.	*R2.*	
	Fator 1 - sensação de esforço	*Factor 1 - sensation of effort*	
	Fator 2 - sensação de ansiedade	*Factor 2 - sensation of anxiety*	
	Fator 3 - sensação de desconforto	*Factor 3 - sensation of discomfort*	
	Dispneia total	*Total dyspnea*	
	VCP.		
	Fator 1 - sensação de esforço		
	Fator 2 - sensação de ansiedade		
	Fator 3 - sensação de desconfortoDispneia total		

**Legenda:** T1 = tradutor 1 inglês para português; T2 = tradutor 2 inglês para português; VCP = versão em português do consenso entre os dois tradutores 1 e 2; R1 = tradutor da retrotradução 1 português para inglês; R2 = tradutor da retrotradução 2 português para inglês

A necessidade de consenso levou a ajustes substanciais, incluindo mudanças no enunciado para garantir clareza e autoexplicação, bem como na formulação das chaves de resposta para assegurar que a escala mantivesse sua precisão e utilidade clínica. Quanto aos itens da escala, foram revisadas questões específicas para melhor correspondência cultural e compreensão global, adaptando termos e contextos temporais para facilitar uma resposta mais precisa e abrangente dos pacientes.

A adaptação e precisão técnica na tradução, aumentam a sensibilidade necessária para considerar diferenças culturais e linguísticas, garantindo que a escala seja eficiente e compreensível em diferentes contextos clínicos e sociais para o Português Brasileiro.

Inicialmente, as chaves de resposta da escala traduzida foram: “Não”, “Um pouco”, “Mais ou menos”, “Muito” e “Demais”. Na aplicação inicial do pré-teste, com 35 pacientes, usando-se as categorias de resposta do protocolo original, não houve nenhum questionamento sobre o conteúdo dos itens, mas sim quanto ao que entendia na opção “Demais”, relacionando a palavra a um contexto de “excesso”. Sendo assim, foi realizada nova discussão com o mesmo comitê de especialistas formado pelos cinco profissionais, ajustando-se a escala de respostas para “Não”, “Um pouco”, “Mais ou menos”, “Bastante” e “Muito”. A escala final com a chave de respostas modificada foi aplicada em mais cinco novos sujeitos, totalizando os 40 indivíduos do estudo, não sendo identificadas nenhuma dificuldade. Não houve marcação na escolha “Não aplicável” o que reforça o reconhecimento fácil dos sintomas relacionados à dispneia, nessa população.

O protocolo original apresenta três fatores e, embora não tenha sido feita análise confirmatória, é interessante apontar que com relação aos escores da Escala de Dispneia Oncológica - CDS-Br, o Fator 1 - Sensação de esforço apresentou uma média de 8,15, o Fator 2 - Sensação de ansiedade apresentou uma média de 4,3, e o Fator 3 - Sensação de desconforto apresentou uma média de 3,4. O escore total da dispneia apresentou uma média de 15,85 (Tabela 2).

**Tabela 2:** Análise descritiva dos escores dos fatores da Escala de Dispneia Oncológica - CDS-Br em indivíduos oncológicos com queixa de dispneia

Variável	Média	DP	Mínimo	Máximo	1Q	Mediana	3Q
Fator 1 - Sensação de esforço	8,13	5,24	0,00	19,00	3,25	7,50	12,00
Fator 2 - Sensação de ansiedade	4,30	3,89	0,00	13,00	1,00	3,00	8,00
Fator 3 - Sensação de desconforto	3,40	3,04	0,00	9,00	0,00	3,00	6,00
Total da dispneia	15,83	10,66	1,00	38,00	7,00	13,00	25,75

Análise descritiva

**Legenda:** DP = desvio padrão; 1Q = primeiro quartil; 3Q = terceiro quartil

A Tabela 3 mostra que não foi possível comparar a proporção de respostas da chave habitual de resposta da Escala de Dispneia Oncológica - CDS-Br com a opção “Não aplicável” para todos itens, visto que todos os indivíduos oncológicos com queixa de dispneia que participaram do pré-teste responderam uma das opções da chave de respostas habitual da Escala de Dispneia Oncológica - CDS-Br, não utilizando a opção “Não aplicável”.

**Tabela 3:** Análise descritiva da comparação da proporção observada e obtida para a comparação das categorias da chave de resposta habitual e não aplicável para cada item da Escala de Dispneia Oncológica - CDS-Br em indivíduos oncológicos com queixa de dispneia

Variável e categorias	n	%	Proporção observada	Proporção do teste
Item 1				
Respostas 1-5	40	100,00	1,00	0,5
Item 2				
Respostas 1-5	40	100,00	1,00	0,5
Item 3				
Respostas 1-5	40	100,00	1,00	0,5
Item 4				
Respostas 1-5	40	100,00	1,00	0,5
Item 5				
Respostas 1-5	40	100,00	1,00	0,5
Item 6				
Respostas 1-5	40	100,00	1,00	0,5
Item 7				
Respostas 1-5	40	100,00	1,00	0,5
Item 8				
Respostas 1-5	40	100,00	1,00	0,5
Item 9				
Respostas 1-5	40	100,00	1,00	0,5
Item 10				
Respostas 1-5	40	100,00	1,00	0,5
Item 11				
Respostas 1-5	40	100,00	1,00	0,5
Item 12				
Respostas 1-5	40	100,00	1,00	0,5

Teste Binominal de uma amostra

**Legenda:** n = frequência absoluta; % = frequência relativa

A Tabela 4 compara a proporção observada e esperada de cada opção de resposta, para cada item da Escala de Dispneia Oncológica - CDS-Br. Não houve diferença para o item 10 (X^2^=2,500), que se refere a questão: “Você sente que suas vias respiratórias estão mais fechadas?” apontando para um padrão de respostas mais aleatório, sem tendência a uma única chave de resposta mais frequente para a questão na população oncológica.

**Tabela 4:** Análise descritiva da comparação da proporção observada e obtida para cada categoria da chave de resposta habitual para cada item da Escala de Dispneia Oncológica - CDS-Br em indivíduos oncológicos com queixa de dispneia

	n Observado	n Esperado	Padrão	X^2^	gl	p-valor
Item 1				14,750	4	0,005
1	2	8,0	-6,0			
2	3	8,0	-5,0			
3	10	8,0	2,0			
4	10	8,0	2,0			
5	15	8,0	7,0			
Total	40					
Item 2				15,500	4	0,004
1	1	8,0	-7,0			
2	4	8,0	-4,0			
3	9	8,0	1,0			
4	11	8,0	3,0			
5	15	8,0	7,0			
Total	40					
Item 3				16,750	4	0,002
1	1	8,0	-7,0			
2	4	8,0	-4,0			
3	9	8,0	1,0			
4	10	8,0	2,0			
5	16	8,0	8,0			
Total	40					
Item 4				13,000	4	0,011
1	11	8,0	3,0			
2	13	8,0	5,0			
3	2	8,0	-6,0			
4	11	8,0	3,0			
5	3	8,0	-5,0			
Total	40					
Item 5				26,750	4	0,000
1	18	8,0	10,0			
2	13	8,0	5,0			
3	6	8,0	-2,0			
4	2	8,0	-6,0			
5	1	8,0	-7,0			
Total	40					
Item 6				17,000	4	0,002
1	6	8,0	-2,0			
2	18	8,0	10,0			
3	4	8,0	-4,0			
4	8	8,0	0,0			
5	4	8,0	-4,0			
Total	40					
Item 7				16,750	4	0,002
1	17	8,0	9,0			
2	5	8,0	-3,0			
3	6	8,0	-2,0			
4	10	8,0	2,0			
5	2	8,0	-6,0			
Total	40					
Item 8				27,000	4	0,000
1	21	8,0	13,0			
2	3	8,0	-5,0			
3	5	8,0	-3,0			
4	6	8,0	-2,0			
5	5	8,0	-3,0			
Total	40					
Item 9				41,250	4	0,000
1	24	8,0	16,0			
2	2	8,0	-6,0			
3	5	8,0	-3,0			
4	6	8,0	-2,0			
5	3	8,0	-5,0			
Total	40					
Item 10				2,500	4	0,645
1	11	8,0	3,0			
2	7	8,0	-1,0			
3	5	8,0	-3,0			
4	9	8,0	1,0			
5	8	8,0	0,0			
Total	40					
Item 11				28,000	4	0,000
1	21	8,0	13,0			
2	7	8,0	-1,0			
3	5	8,0	-3,0			
4	5	8,0	-3,0			
5	2	8,0	-6,0			
Total	40					
Item 12				9,500	4	0,050
1	15	8,0	7,0			
2	5	8,0	-3,0			
3	4	8,0	-4,0			
4	7	8,0	-1,0			
5	9	8,0	1,0			
Total	40					

Teste Qui-Quadrado de uma amostra

**Legenda:** n = frequência absoluta; X^2^ = qui-quadrado; gl = graus de liberdade

A composição final da versão brasileira traduzida e transculturalmente adaptada da *The Cancer Dyspnoea Scale - CDS* ^(6)^, chamada de Escala de Dispneia Oncológica - CDS-Br (Apêndice 1), apresenta 12 itens, como o protocolo original.

## DISCUSSÃO

A dispneia é uma experiência subjetiva de desconforto respiratório que consiste em sensações qualitativamente distintas que variam em intensidade. Nos pacientes oncológicos, é um dos sintomas mais comuns e angustiantes agravado por outros sintomas relacionados, como a fadiga e a ansiedade, resultando em limitação funcional^(8)^. Diversas funções podem sofrer prejuízos importantes pela alteração respiratória, sendo esses diretamente acarretados pela dispneia ou por seu impacto na evolução prognóstica^(6,8)^. A dispneia pode interferir na produção vocal devido às alterações no fluxo respiratório, prejudicando a qualidade de comunicação. Em todos os cenários possíveis, a dispneia e suas morbidades podem comprometer negativamente a qualidade de vida dos pacientes oncológicos^(7)^.

Devido à complexidade e subjetividade da dispneia, os profissionais de saúde precisam ter atenção ao manejo do quadro para garantir a abordagem adequada e definir a indicação correta nas intervenções de aspectos relacionados que interferem diretamente no quadro respiratório. Os protocolos de autoavaliação auxiliam os profissionais a entenderem melhor o impacto da dispneia nos pacientes e também colaboram para que o paciente compreenda de forma clara de que maneira essa alteração tem afetado seu bem-estar^(6)^. No Brasil, não existem escalas ou questionários que avaliem a dispneia especificamente de pacientes oncológicos, a partir da autoavaliação, levando em consideração os fatores multidimensionais da dispneia.

A *The Cancer Dyspnoea Scale - CDS* ^(6)^ é um questionário breve de autoavaliação que consiste em três fatores e 12 itens. É a primeira escala a abordar a natureza multidimensional da dispneia do paciente oncológico, focando diretamente na experiência da dispneia em si, no lugar de avaliar apenas o esforço físico que pode desencadeá-la. É uma ferramenta única, autoaplicável, validada e comprovadamente confiável para a avaliação da dispneia em pacientes oncológicos. As pontuações totais e por subfator do *The Cancer Dyspnoea Scale - CDS* refletem a intensidade da dispneia e cada subfator captura diferentes aspectos da dispneia, correlacionando-se com o estado físico e o estado psicológico^(6)^.

A fase inicial do processo de validação de um questionário é a tradução e adaptação transcultural, fundamental para viabilizar o uso desse instrumento em uma língua e cultura distintas daquelas em que foi originalmente concebido, devendo seguir diretrizes que garantam o rigor científico desse procedimento^(12)^. Durante as fases de síntese e consenso entre os especialistas, foram feitas modificações para tornar as perguntas da escala mais claras e simples, juntamente com os itens de marcação, de modo a garantir que fossem compreensíveis para diferentes grupos sociais e culturais no Brasil. Foi necessário simplificar algumas perguntas e ajustar termos que não afetavam o significado geral das frases quando comparado com o instrumento original.

Na fase de pré-teste, verificou-se que todas as questões da escala eram compreensíveis. Nenhum participante deixou uma pergunta sem resposta, questionou seu significado ou, quando foi oferecida a marcação da opção “Não aplicável”, ela não foi utilizada. Contudo, houve margem para discussão em relação à última opção da chave de respostas proposta originalmente (“Não”, “Um pouco”, “Mais ou menos”, “Muito” e “Demais”). Alguns participantes referiram que “Demais” poderia ser um fator de alteração, sendo assim a escala foi rediscutida entre os especialistas e ajustada para os termos propostos. Nessa versão, não houve dúvidas na autoavaliação dos novos participantes incluídos.

Com relação a proporção observada para cada opção de resposta, nota-se diferença estatisticamente significativa em todos os itens, com exceção do item 10 (“Você sente que suas vias respiratórias estão mais fechadas?”). Devido aos diagnósticos oncológicos dos participantes é possível relacionar o padrão de respostas ao fato da similaridade entre os sujeitos do grupo. Com relação ao item 10, deve-se levar em consideração que essa questão pode sofrer interferência com relação ao meio respiratório do participante. Participaram do estudo pacientes em uso de traqueostomia protetiva, traqueostomia por obstrução de vias aéreas, traqueostomia permanente e também com pacientes sem uso do dispositivo. A via respiratória pode ter colaborado para o padrão de respostas desse item, o que demonstra a efetividade do uso da escala na prática clínica para diferentes tipos de pacientes, já que sugere que as múltiplas dimensões da dispneia se sobrepõem de maneira complexa e estão intimamente relacionadas^(6)^.

A aplicação do CDS-Br mostrou-se viável e prática em ambiente clínico. A simplicidade e facilidade de preenchimento por pacientes oncológicos com dispneia são características cruciais do instrumento^(6)^.

A adaptação transcultural realizada neste estudo resultou na versão da Escala de Dispneia Oncológica - CDS-Br, traduzida e transculturalmente adaptada para o Português Brasileiro, oferecendo um instrumento único de autoavaliação da dispneia para pacientes oncológicos.

## CONCLUSÃO

A versão traduzida e transculturalmente adaptada para o Português Brasileiro foi denominada Escala de Dispneia Oncológica - CDS-Br. No processo de tradução e adaptação transcultural foram necessários ajustes para que seja aplicável ao Português Brasileiro, refletindo a versão original. A Escala de Dispneia Oncológica - CDS-Br mostrou ser um instrumento de fácil aplicação e compreensão para a autoavaliação da dispneia de pacientes oncológicos.

## Apêndice 1 Escala de Dispneia Oncológica - CDS-Br, versão traduzida e culturalmente adaptada da *The Cancer Dyspnoea Scale - CDS* ^(6)^

**Table:**

**ESCALA DE DISPNEIA ONCOLÓGICA - CDS-Br**					
Gostaríamos de lhe perguntar sobre sua falta de ar ou dificuldade de respirar. Por favor, responda a cada pergunta marcando o número que melhor descreve sua dificuldade de respirar, nos últimos dias. Responda de acordo com sua primeira impressão.					
	Não	Um pouco	Mais ou menos	Bastante	Muito
1. Você consegue inspirar com facilidade?	1	2	3	4	5
2. Você consegue expirar com facilidade?	1	2	3	4	5
3. Você consegue respirar lentamente?	1	2	3	4	5
4. Você sente falta de ar?	1	2	3	4	5
5. Você transpira e tem palpitações quando está difícil respirar?	1	2	3	4	5
6. Você fica ofegante?	1	2	3	4	5
7. Você tem dificuldade de respirar e fica sem saber o que fazer?	1	2	3	4	5
8. Você acha que sua respiração é superficial?	1	2	3	4	5
9. Você sente que sua respiração pode se interromper?	1	2	3	4	5
10. Você sente que suas vias respiratórias estão mais fechadas?	1	2	3	4	5
11. Você acha que pode se sufocar?	1	2	3	4	5
12. Você sente que tem algo limitando sua respiração	1	2	3	4	5
**Como calcular os resultados**					
**1.** Some as pontuações de cada fator.					
Fator 1 = (itens 4 + 6 + 8 + 10 + 12) – 5 = **sensação de esforço**					
Fator 2 = (itens 5 + 7 + 9 + 11) – 4 = **sensação de ansiedade**					
Fator 3 = 15 – (itens 1 + 2 + 3) = **sensação de desconforto**					
**2.** Some a pontuação total de cada fator = **dispneia total**					
As subtrações servem para fazer ajustes para 0 corresponder ao estado de ausência de dispneia.
